# Detection of tem-1 and class-1 integrons in multidrug resistant uropathogens from HIV patients with asymptomatic bacteriuria in a Tertiary Care Hospital, SouthWest Nigeria

**DOI:** 10.4314/ahs.v22i1.56

**Published:** 2022-03

**Authors:** Olubisi Ajala, Babatunde Odetoyin, Temilola Owojuyigbe, Adebola Onanuga

**Affiliations:** 1 Department of Medical Microbiology and Parasitology, Obafemi Awolowo University, Ile-Ife, Nigeria; 2 Department of Haematology and Immunology, Obafemi Awolowo University, Ile-Ife, Nigeria; 3 Department of Pharmaceutical Microbiology and Biotechnology, University of Maiduguri, Maiduguri, Borno State, Nigeria

**Keywords:** Antimicrobial resistance, TEM 1, asymptomatic bacteriuria, HIV, uropathogens, Class 1 integrons

## Abstract

**Background:**

Human immunodeficiency virus (HIV) infected individuals are at increased risk of asymptomatic bacteriuria (ASB) due to immune suppression. The increasing resistance of uropathogens necessitates the need for regular monitoring of their profile to reduce drug resistance.

**Objectives:**

We determined the prevalence of ASB and the characteristics of antibiotic-resistant uropathogens isolated from HIV patients.

**Methods:**

Mid-stream urine samples from 100 HIV positive and 100 HIV negative healthy individuals were cultured for significant bacteriuria. The isolates were identified by standard techniques and their susceptibility patterns determined by the Kirby-Bauer disc diffusion technique. All the Gram-negative isolates were screened for ESBL production by combined disc method, ESBL genes and class 1 integrons by Polymerase chain reaction.

**Results:**

Nine (9%) HIV positive individuals and 4 (4%) healthy individuals had ASB yielding a total of 13 (6.5%) uropathogens dominated by *Escherichia coli* (53.9%). All isolates were multidrug resistant. Five isolates harboured both the blaTEM-1 gene and class 1integrons while *Serratia liquefaciens* produced ampC.

**Conclusion:**

There is a higher burden of ASB characterized by multi-drug resistant uropathogens among HIV patients. Thus emphasizing the need for continuous resistance surveillance and antibiotic stewardship in our environment to reduce drug resistance and prevent treatment failure.

## Introduction

The Acquired immune deficiency syndrome caused by the human immunodeficiency virus (HIV) is a major global health problem 1. According to the 2018 UNAIDS reports, 1.9 million people with HIV live in Nigeria. Of these, 67% knew their status, 53% were on treatment and 42% were virally suppressed 2. Although there has been a decline in the number of AIDS-related deaths since 2010, with a 26% fall, from 72,000 to 53,000 deaths, the number of new HIV infections has increased within the same period from 120,000 to 130,000. Even with the widespread HIV awareness programme, many people still do not know their status; they either go on with their lives undiagnosed or present with multiple infections later in life 2.

HIV infected individuals are at higher risk of infections due to the alteration of their normal defence by the virus 3. High viral load (≥100,000 copies/ml) promotes the danger of opportunistic infections 4. In individuals with high viral load, bladder areflexia and hyporeflexia are regular neurologic complications, which result in urinary stasis and eventually urinary tract infections (UTIs) 5.

Urinary tract infections may be symptomatic or asymptomatic; whether asymptomatic bacteriuria (ASB) precedes symptomatic UTI in people with HIV is not clearly understood. ASB is the presence of at least 10^5^ CFU/ml of one or two uropathogens in a culture of mid-stream urine collected from a patient without symptoms of UTI 6. While some studies showed no significant differences in the prevalence of ASB between HIV positive and HIV negative populations, majority of other studies reported a higher prevalence particularly among women and those with high viral load 7–10. Associated risk factors for ASB include gender, marital status and high viral load/low CD4 count^8^–^10^. Although, several investigators have looked at the problem of ASB among individuals with HIV in different populations, there are still unanswered questions 9. ASB can lead to adverse outcomes in pregnant women and patients with urological disorders, however, it is unknown if this so in the HIV population 11,12.

Among the group of antibiotics commonly used to treat UTIs, β-lactams are the most extensively used agents 13. However, the emergence of resistance to these agents has become a global problem due to the irrational use of antibiotics at both community and hospital levels. In the last ten years, there has been an increasing trend of multidrug-resistant uropathogens particularly ESBL-producing uropathogens worldwide, including in Nigeria 14,15. ESBLs are enzymes that hydrolyzes penicillins, oxyimino-cephalosporins, and monobactams 16. The specific ESBL-producing strains have different genetic characteristics, which may be responsible for specific characteristics. Most ESBLs originate from the widespread broad-spectrum beta-lactamases SHV-1 and TEM-1 through definite mutations. Nevertheless, their prevalence and occurrence vary significantly in different geographical areas 17.

The determinants of antibiotic resistance are normally transferred among bacterial strains by mobile genetic elements, including integrons 18. Integrons integrate and disseminate resistance genes among bacteria. To date, many classes of integrons have been described based on the sequences of their integrase genes (intI) 19. Of these, classes 1 and 2 integrons are the most frequently associated with antibiotic resistance in Gram-negative bacteria 18.

Assessment of agents of UTI and their susceptibility profile is imperative as part of global efforts to contain the emergence and dissemination of antimicrobial resistance. Thus, we investigated the prevalence of ASB and antimicrobial resistance characteristics of uropathogens isolated from HIV patients.

## Materials and methods

### Study Area and Study Population

This study involved 100 HIV positive individuals attending the Virology research clinic of Obafemi Awolowo University Teaching Hospitals Complex (OAUTHC), Ile-Ife, Nigeria. Sampling was performed over threemonths, and midstream urine samples were aseptically collected consecutively from patients aged 18 years and above as they presented to the clinic. A control group of 100 healthy HIV negative blood donors of the same age range were also recruited at the Department of Haematology and Blood Transfusion of the hospital. Those with conditions that can predispose them to UTIs and those with symptoms of UTI and on antibiotics within the preceding 14 days of sampling were excluded from the study.

The patients' information was obtained using a pro forma. Ethical approval was obtained from the Ethics and Research Committee of OAUTHC to conduct the study. Written informed consent was required to participate in the study. The samples were collected from participants in sterile universal bottles and transported on ice to the laboratory for investigations within two hours of collection.

### Processing of Samples

The samples (5mL) were centrifuged at 2500 rpm for five minutes, and urine sediments were examined under the microscope for casts, crystals, red blood cells, leukocytes, and bacteria. Well-mixed uncentrifuged specimens were cultured within two hours of collection on Cysteine Lactose Electrolyte Deficient Medium (CLED) (HiMedia, India) and blood agar using a standard calibrated (0.01mL) platinum wire loop. Each culture plate was read after 18–24 hours incubation at 37°C under aerobic conditions. Samples that yielded one isolate with a colony count ≥ 10^5^ CFU/mL or two isolates with one or both colonies count ≥10^5^ CFU/mL were considered to have significant bacteriuria. Samples with counts <10^5^ CFU/mL were not significant. Bacterial isolates were identified based on their morphology, Gram's staining reaction, and biochemical characteristics. The Gram-negative isolates were characterized with Microbact™ GNB 24E identification kit (Oxoid Ltd, Basingstoke, United Kingdom) and identified with Microbact™ Computer-Aided Identification Package, version 2.04 (Oxoid Ltd, Basingstoke, United Kingdom).

### Antibiotics Susceptibility Testing

Bacterial susceptibility to 20 antibiotics was determined using the Kirby-Bauer disc diffusion method following the guidelines of the Clinical and Laboratory Standard Institute (CLSI)^20^. The antibiotics used were: nitrofurantoin (300µg), gentamicin (10µg), amoxicillin-clavulanate (30µg), ceftriaxone (30µg), cefotaxime (30µg), sulfamethoxazole-trimethoprim (25µg), chloramphenicol (30µg), ciprofloxacin (5µg), meropenem (10µg), ceftazidime (30µg), penicillin (10U), ofloxacin (5µg), cefepime (30µg), ampicillin (10µg), tetracycline (30µg), streptomycin (10µg), erythromycin (15µg), and cefoxitin (30µg), aztreonam (30µg). *Staphylococcus aureus* ATCC25923 and *Escherichia coli* ATCC 25922 were used as reference strains for the test. The minimum inhibitory concentration (MIC) for vancomycin was determined using the microtitre dilution method according to the recommendation of CLSI 20 The Multiple Antibiotic Resistance (MAR) index was calculated as described by Krumperman 21. Isolates with MAR index values greater than 0.2 were considered to have originated from high-risk sources where antibiotics were frequently used while a MAR index values of less than or equal to 0.2 indicates that the isolate originates from low-risk sources where antibiotics are rarely or never used. Multidrug resistance (MDR) was defined as non-susceptibility to at least one agent in three or more categories of antimicrobial agents 22.

Cefpodoxime 10µg discs with and without clavulanic acid (2.5µg) were used to confirm ESBL production by the combination disc method. Cefpodoxime 10µg with and without boronic acid (400µg) (AmpC inhibitor) were used to confirm AmpC production. An increase in the inhibition zone diameter of > 5 mm in a third-generation cephalosporin disc combined with clavulanic acid or boronic acid, compared with the third-generation cephalosporin alone, indicated ESBL production or AmpC production respectively 23. The results were interpreted using the Multichrome Antibiotic Susceptibility Test (MAST) disc calculator (Mast group Ltd, England).

### DNA extraction

The DNA of the Gram-negative isolates was extracted using the boiling method 24. Three colonies of each isolate were emulsified in 100µl of sterile distilled water in a clean Eppendorf tube, boiled for 15 minutes and centrifuged at 10,000 rpm for five minutes in a microcentrifuge. The supernatant was transferred to a new Eppendorf tube after centrifugation and was used as template DNA for polymerase chain reaction (PCR).

### Amplification of extended-spectrum β-lactamase genes and Class 1 integrons

All the Gram-negative isolates that were resistant to ampicillin but not confirmed as ESBL producers with the combination discs were screened by PCR using primers specific for the detection of blaSHV, blaTEM and blaCTX-M genes as described by Bebe et al. 14 (Table 1). A 25µl reaction mix composed of 12.5 µl one Taq Quick-Load 2X master mix with standard buffer, 0.5µl of 10µM each of forward primer and reverse primer, 3µl template DNA and 8.5µl of nuclease-free water. Amplification reactions involved initial denaturation at 94°C for 5 minutes, 35 cycles of denaturation at 94°C for 1 minute, annealing at 45°C for 30 seconds, and extension at 72°C for 1 minute, and a final extension at 72°C for 5 minutes. *E. coli* ATCC 25922 was used as a negative control.

**Table 1:** Primers Used for Amplification of Resistance Genes and Class 1 Integrons by Polymerase Chain Reaction

Gene	Primer	Sequence (5′-3′)	Size(bp)	T°C
SHV	SHV-F	CGCCTGTGTATTATCTCCCT	293	45
	SHV-R	CGAGTAGTCCACCAGATCCT		
TEM	TEM-F	TTTCGTGTCGCCCTTATTCC	403	45
	TEM-R	ATCGTTGTCAGAAGTAAGTTGG		
CTX-M	CTX-M-F	CGCTGTTGTTAGGAAGTGTG	874	45
	CTX-M-R	GGCTGGGTGAAGTAAGTGAC		
Class 1 Integrons	Lévesque5CS	GGC ATC CAA GCA GCA AG	variable	50
	Lévesque3CS	AAG CAG ACT TGA CCT GA		

Also, the isolates were screened for class 1 integrons by PCR using Levesque 5CS and 3CS primers 25 (Table 1). The reaction mix for each strain was constituted as follows: 12.5µl one Taq Quick-Load 2X master mix with standard buffer, 0.5µl of 10µM each of forward primer and reverse primer, 3µl template DNA and 8.5µl of nuclease-free water. Amplification reactions involved initial denaturation at 94°C for 5 minutes, 40 cycles of denaturation at 94°C for 1 minute, annealing at 50°C for 30 seconds, and extension at 72°C for 1 minute, and a final extension at 72°C for 10 minutes in GenAmp® PCR system 9700 (Applied Biosystems). Each amplicon (10µL) was electrophoresed on a 1.5% agarose gel pre stained with 0.5µg/mL Ethidium bromide in 1X Tris-Borate-EDTA (TBE) buffer and viewed with a UVitec transilluminator (Avebury, Cambridge UK). The position of amplified products was estimated by the position of the ladder (Biolab, England). The TEM genes were sequenced using the Nimagen, Brilliant-Dye™ Terminator Cycle Sequencing Kit V3.1, BRD3-100/1000 according to manufacturer's instructions 26.

Sequence chromatogram analysis was performed using Finch TV analysis software version 1.4.0.

### Data Analysis

R statistical Package version 1.2.5033 was used for data analysis 27. Data distribution was assessed using Fisher's Exact Test to compare case and control groups for similarity. Association or non-association of parameters and inferences were based on the generated 2-sided P-values. A p-value less than or equals to 0.05 was considered significant.

## Results

Demographic Characteristics of the Study Participants One hundred HIV infected individuals and 100 HIV negative healthy individuals were recruited into the study from July to October 2019. Both subjects and controls were similar in sex distribution, mean age but different educational levels (Table 2).

**Table 2:** Demographic Characteristics of the Study Participants

Variable	Number Examined	HIV positive n (%)	HIV negative n (%)
**Age group**			
18–33	36	18(50.0)	18(50.0)
34–49	104	53(51.0)	51(49.0)
50–65	60	29(48.3)	31(51.7)
Mean±SD		42.53±10.29	41.01±11.86
**Gender**			
Male	40	20(50.0)	20(50.0)
Female	160	80(50.0)	80(50.0)
**Marital Status**			
Married	153	89(58.2)	64(41.8)
Single	47	11(23.4)	36(76.6)
**Occupation**			
Civil Servant	37	9(24.3)	28(75.7)
Self Employed	92	62(67.4)	30(32.3)
Artisan	28	24(85.7)	4(14.8)
Unemployed	43	5(11.6)	38(88.4)
**Religion**			
Christianity	154	66(42.9)	88(57.1)
Islam	46	34(73.9)	12(26.1)
**Marriage pattern**			
Monogamy	154	89(57.8)	65(42.2)
Polygamy	2	1(50.0)	1(50.0)
**Education Level**			
No formal	2	2(100.0)	0(0.0)
Primary	13	12(92.3)	1(7.7)
Secondary	103	66(64.1)	37(35.9)
Tertiary	82	20(24.4)	62(75.6)

## Prevalence of Asymptomatic Bacteriuria in the Study Population

Nine (9%) HIV positive individuals and 4 (4%) HIV-negative individuals had ASB. All positive cases were found in females in both groups (Table 3). Patients in the age range of 34–49 years had the highest prevalence of ASB (n=4; 44.4%) while those between 50–65 years had the lowest (n=2; 22.2%). There was no significant association between age group and ASB (p=0.5134). Six (66.7%) married patients had ASB compared to 3 (33.3%) that were single. The prevalence of ASB was significantly higher among the married compared with the single (p=0.05). One (11.1%) patients with primary, 6 (66.7%) patients with secondary and 2 (22.2%) patients with tertiary education had ASB respectively. Six (100%) patients in monogamy and none (0%) in polygamy had ASB. There was no relationship between type of marriage and the prevalence of ASB (p>0.05) (Table 4). Patients with a viral load between 20–100 had the highest prevalence of ASB (n=5; 55.6%) while those with a viral load above 100 had the least prevalence of ASB (n=1; 11.1%). There was no significant association between the prevalence of ASB and viral load (p>0.05).

**Table 3:** Prevalence of asymptomatic bacteriuria in the study population

Parameter	Subject		Control	
	
	Frequency (Total)	%	Frequency (Total)	%
**ASB prevalence**				
ASB prevalence in males	0 (20)	0.0	0 (20)	0.0
ASB Prevalance in Females	9 (80)	11.25	4 (80)	5.0
Overall ASB prevalence	9 (100)	9.0	4 (100)	4.0

**Table 4:** Prevalence of ASB in Relation to Gender, Age group, Marital status and Educational level

Parameter	ASB		Total	p-value^a^

	Yes (%) N=9	No (%) N=91	No (%) N=100	
**Gender**				0.198
Female	9 (100)	71 (78.0)	80 (80.0)	
Male	0 (0.0)	20 (22.0)	20 (20.0)	
**Age group**				0.513
18–33	3 (33.3)	15 (16.5)	18 (18.0)	
34–49	4 (44.4)	49 (53.8)	53 (53.0)	
50–65	2 (22.2)	27 (29.7)	29 (29.0)	
**Marital status**				0.05
Married	6 (66.7)	83 (91.2)	89 (89.0)	
Single	3 (33.3)	8 (8.8)	11 (11.0)	
**Religion**				1.000
Christian	6 (66.7)	60 (65.9)	66(66.0)	
Muslim	3 (33.3)	31 (34.1)	34(34.0)	
**Education level**				1.000
No formal	0 (0.0)	2 (2.2)	2 (2.0)	
Primary	1 (11.1)	11 (121)	12 (12.0)	
Secondary	6 (66.7)	60 (65.9)	66 (66.0)	
Tertiary	2 (22.2)	18 (19.8)	20 (20.0)	
**Occupation**				
Civil servant	2 (22.2)	7(7.7)	9 (9.0)	0.2796
Self employed	4 (44.4)	59(64.8)	63 (63.0)	
Artisan	3 (33.3)	20(22.0)	23 (23.0)	
Unemployed	0 (0.0)	5 (5.5)	5 (5.0)	
**Marriage**				1.000
Monogamy	6/6 (100.0)	82/89(98.8)	88(88.0)	
Polygamy	0/6 (0.0)	1/89 (1.2)	1 (1.0)	
**Viral load**				0.547
< 20	3(33.3)	17(18.7)	20	
20–100	5(55.6)	57(62.6)	62	
>100	1(11.1)	17(18.7)	18	

a Fisher's exact test

## Isolated Uropathogens from HIV positive and HIV-Negative Individuals

Thirteen uropathogens were isolated from both groups, comprising nine from HIV positive individuals and four from HIV negative individual. Most isolates were Gram-negative bacilli. *Escherichia coli* preponderated among the isolates 7(53.9%). Other bacteria isolated included *Staphylococcus aureus* (n=1; 7.7%), *Enterobacter agglomerrans* (n=2; 15.4%), and *Staphylococcus epidermidis* (n=1; 7.7%). *Klebsiella oxytoca* (n=1; 7.7%) and *Serratia liquefaciens* (n=1; 7.7%) were isolated only from HIV negative individuals (Table 5).

**Table 5:** Gene Variant, Integrons and Resistance Patterns of Isolated Uropathogens

S/N	Name of Organism	No of Antibiotics Class Used	No to which Isolates Were resistant	MAR Index	Gene	Gene Variant	Class 1 Integrons	Sizes of variable regions in Class 1 Integrons	AmpC	Resistance Pattern
**HIV Positive Individuals**										
S2	*Staphylococcus* *aureus*	13	3	0.3	ND	ND	ND	ND	ND	CXM, SXT, FEP, CRO, CTX, CAZ
S87	*Staphylococcus* *epidermidis*	13	3	0.5	ND	ND	ND	ND	ND	CXM, ERY, NIT,
S12	*Escherichia coli*	13	7	0.4	TEM	TEM 1	YES (TWO)	400bp/1100bp	NO	AMP, PEN, ERY, OFL, CIP, GEN, CXM, FOX, SXT, FEP
S69	*Escherichia coli*	13	7	0.5	TEM	TEM1	YES (TWO)	400bp/1700bp	NO	AMP, PEN, ERY, GEN,CXM, CHL,SXT, TET, CEFP
S75	*Enterobacter* *agglomerans* *complex*	13	7	0.4	TEM	TEM 1	NONE	NA	NO	AMP, PEN, ERY, NIT, CIP, GEN, CXM, SXT, CTX
S81	*Enterobacter* *agglomerans* *complex*	13	6	0.5	TEM	TEM 1	YES (THREE)	400bp/1000bp/1900bp	NO	AMP, PEN, ERY, OFL, CIP, GEN, CHL, SXT
S85	*Escherichia coli*	13	7	0.3	NEG	ND	ND	NA	ND	CIP, GEN, CXM, SXT, ERY, FEP, CRO, CTX, AMP, PEN, CEFP
S92	*Escherichia coli*	13	7	0.4	NEG	ND	ND	NA	ND	CIP, GEN, CXM, FOX, ERY,AMP, PEN
S99	*Escherichia coli*	13	3	0.2	NEG	ND	ND	NA	ND	GEN, ERY, AMP, PEN
**HIV Negative Individuals**										
C16	*Escherichia coli*	13	7	0.4	TEM	TEM 1	NONE	NA	ND	GEN, CXM, SXT, ERY, FEP, CRO, AMP, PEN
C29	*Klebsiella* *oxytoca*	13	2	0.2	TEM	TEM 1	YES (THREE)	400bp/1500bp/1600bp	NO	AMP, PEN, ERY
C46	*Serratia* *liquefaciens* *complex*	13	6	0.4	TEM	TEM 1	NONE	NONE	YES	AMP, PEN, ERY, GEN, CXM, FOX, AUG
C98	*Escherichia coli*	13	6	0.4	TEM	TEM 1	YES (FOUR)	400bp/1000bp/1900bp/2500bp	NO	AMP, PEN, ERY, OFL, CIP, GEN, SXT

OFL= Ofloxacin, NIT= Nitrofurantoin, CIP= Ciprofloxacin, GEN= gentamicin, CXM= Cefuroxime, CHL= Chloramphenicol, FOX= Cefoxitin, SXT= Sulphamethoxazole-trimethoprim, ERY= Erythromycin, FEP= Cefepime; CRO= Ceftriaxone, TET= Tetracycline, CTX= Cefotaxime, CAZ= Ceftazidime, MEM= Meropenem, AUG= Amoxicillin-clavulanate, AMP= Ampicillin, ATM= Aztreonam, PEN= Penicillin, VAN= Vancomycin, OXA= Oxacillin CEFP=Cefpodoxime, NA= Not applicable, ND= Not determine

## Antibiotic Resistance Patterns of Isolated Uropathogens

All the isolates were sensitive to meropenem. The Gram-positive and Gram-negative isolates were resistant to cefuroxime and ampicillin respectively. *S. aureus* was resistant to cefepime and cefotaxime while S. epidermidis was resistant to nitrofurantoin, erythromycin, sulphamethoxazole-trimethoprim. All Gram-negative isolates were susceptible to ceftazidime, meropenem, and aztreonam (Table 5).

The multiple antibiotic resistance (MAR) index ranges from 0.2 – 0.5. All uropathogens were isolated from high-risk sources (MAR index >0.2). The highest number of classes of antibiotics to which an organism was resistant to was seven while the lowest was two (Table 5). The two isolates of *E. agglomerans* from the subjects were resistant to seven and six classes of antibiotics respectively while *S. epidermidis* isolated from the subject was resistant to three classes. The seven isolates of *E. coli* from both subject and control were resistant to 3–7 classes (Table 5).

## Detection of AmpC, TEM I and Class 1 Integrons

**Figure 1:**
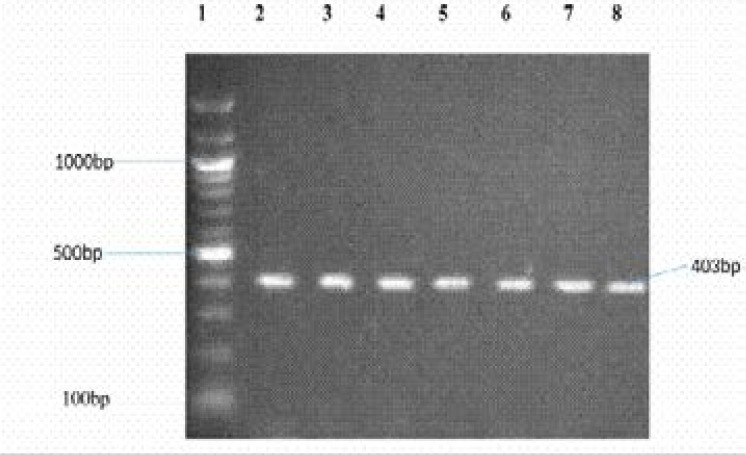
PCR Amplification of TEM (403bp) Lane 1: 100bp Ladder; Lane 2: S81- *Enterobacter agglomerans complex*; Lane 3: S75- *Enterobacter agglomerans complex*; Lane 4: C46- *Serratia liquefaciens complex*; Lane 5: S69- *Escherichia coli*; Lane 6: S12- *Escherichia coli*; Lane 7: C98- *Escherichia coli*; Lane 8: C16- *Escherichia coli*

**Figure 2:**
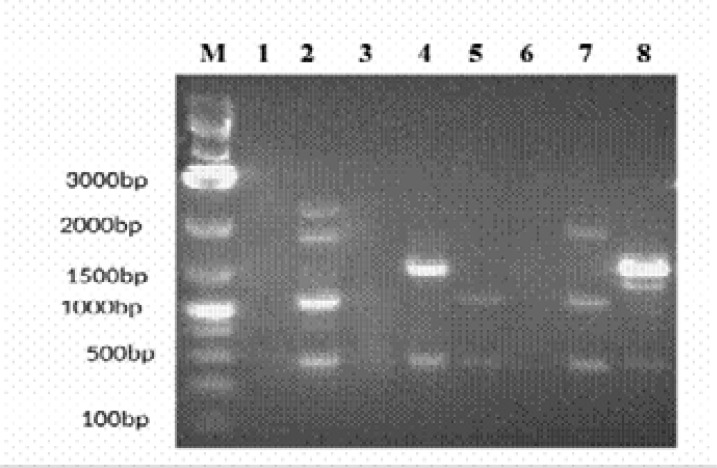
**Gel picture of Class I Integrons** Lane M: 1kb+ ladder; Lane 1: C16- *Escherichia coli*; Lane 2: C98-*Escherichia coli;* Lane 3: S75-*Enterobacter agglomerans*; Lane 4: S69- *Escherichia coli*; Lane 5: S12-*Escherichia coli*; Lane 6: C46-*Serratia liquefaciens*; Lane 7:S81-*Enterobacter agglomerans complex*; Lane 8: C29-*Klebsiella oxytoca*

None of the Gram-negative isolates was an ESBL producer. AmpC was detected only in *Serratia liquefaciens*. Eight isolates harboured the TEM 1 gene (Figure 1). Five of the eight isolates with TEM1 harboured class 1 integrons with different variable regions (Figure 2) (Table 5).

## Discussion

We observed a higher prevalence of ASB in patients infected with HIV (9%) compared to HIV-negative individuals (4%) in our study. The prevalence of ASB as observed is similar to the prevalence of 6.0–25% reported in previous studies 8,28,29. The higher prevalence of ASB in HIV patients may be attributed to immunosuppression 3.

Contrary to reports that high viral load promotes infections in HIV patients 30,31, we did not find any significant association between the prevalence of ASB and viral load. This may be because all the patients were on an antiretroviral drug when the study was conducted. All positive cases of ASB were found only in the females in both groups. ASB was more prevalent among the females and highly significant among the married.. The higher prevalence of bacteriuria in the females could be attributed to the shortness of their urinary tracts 28.

*E. coli* was the commonest isolate (53.9%) followed by Enterobacter agglomerans (15.4%). ASB aetiology varies from one location to another and with patients ‘conditions. Globally, *E. coli* is the commonest uropathogens implicated in ASB 32,33. Previous studies in Nigeria have also reported it as the commonest pathogen implicated in ASB 28,34. Consequently, its dominance is in tandem with the previous reports. We isolated two strains of E. agglomearans exclusively from HIV patients. *E. agglomearans* is an opportunistic pathogen that has been implicated in hospital-acquired infections mostly in immunocompromised individuals 35. Hence, its isolation from HIV patients corroborates their impaired immune status 36. Isolation of *S. aureus* and *S epidermidis* is not limited to our study. Previous studies in the study environment and across the globe similarly reported them as frequently isolated uropathogens 14,30,34. Isolation of these bacteria may be because they are mostly normal skin flora and can be accidentally introduced to the urethra during sexual intercourse 37.

Multi-drug resistant (MDR) bacteria continue to be a problem in hospitalized patients worldwide. The frequency of MDR among clinical isolates varies globally and in different geographic locations and is rapidly changing with time 22. The MAR index ranges of 0.2 – 0.5 observed in this study implies that the uropathogens were isolated from sources where they make use of antibiotics frequently. The rates of resistance we observed in our study are similar to that of other studies in other developing countries 30,38. All the isolates were susceptible to meropenem. Apart from meropenem, cefoxitin, vancomycin, oxacillin, augmentin, chloramphenicol, penicillin, ampicillin and tetracycline were also active against the Gram-positive bacteria. However, all the Gram-positive and negative bacteria were resistant to cefuroxime and ampicillin respectively. Also, most of the Gram-negative isolates were resistant to cotrimoxazole (n=8; 61.6%) which agrees with the previous studies on ASB in the HIV population 7,39. Since the use of cotrimoxazole as prophylaxis to prevent opportunistic infections in HIV Infected individuals, resistance to it has increased sharply. A recent study by Aina and Olajuyigbe 40 in Nigeria showed that prolong use of cotrimoxazole is associated with the rapid development of resistance to the antibiotic. Other investigators have also reported this trend in HIV patients that were on cotrimoxazole prophylaxis 39,41. Hence, high resistance to cotrimoxazole in HIV patients in our study may be due to its use as prophylaxis against opportunistic infections in this environment. A high percentage (71.4%) of the Gram-negative isolates from HIV patients was resistant to ciprofloxacin. Globally, resistance to fluoroquinolones by uropathogens has been increasing. According to the report of the Study for the monitoring of antimicrobial resistance trends (SMART) that investigated fluoroquinolone resistance in Gram-negative uropathogens worldwide, the rates of fluoroquinolone resistance varied widely across countries with a range of 6% to 75% 42. Studies across Nigeria have also reported different rates of fluoroquinolone resistance up to 50% which is comparable to the present study 43,44. Unregulated use of fluoroquinolones in Nigeria has probably led to a low degree of susceptibility of uropathogens to this drug. Hence, rational use of this drug may allow it to recover its potency.

None of the Gram negative isolates was positive to the phenotypic ESBL test but eight isolates (72.2%) were positive for blaTEM-1 gene. TEM-1 is the most prevalent β-lactamase in Gram-negative bacteria and is commonly found on conjugative plasmids which contribute to its spread in bacteria. TEM-1 beta-lactamase encodes resistance to penicillin, ampicillin and first-generation cephalosporins 16. Although TEM-1 only encodes resistance to penicillins and early cephalosporins, the resistance of its variants has surpassed second-, third-, and fourth-generation cephalosporins, and monobactams^16^. TEM-1 and TEM-2 penicillinases are the evolutionary precursors of the TEM family which is the largest and extensively circulated group of these enzymes of which TEM-1 is encoded by series of gene alleles, blaTEM-1A to blaTEM-1F which differ from each other by definite silent mutations 45. This implies that isolates that harbor TEM-1 genes can serve as reservoirs of ESBL genes that may emerge through mutations. Thus, the detection of TEM-1 genes in Gram-negative bacteria should be included as one of the strategies for preventing the spread of ESBLs producing organisms.

Furthermore, five of the eight isolates (62.5%) with TEM-1 genes harboured Class 1 integrons with variable regions ranging from 400bp to 2500bp. Integrons are reservoirs of antimicrobial resistance genes in bacteria^25^. The resistance genes are found in their variable regions as cassettes, and might encode resistance to many antibiotics 25. Until date, more than 8000 gene cassettes have been identified and they encoded resistance to almost all antibiotics 46. Although, we did not characterize the gene cassettes of the identified integrons, however, their presence in some isolates with TEM 1 may contribute to its dissemination and the multidrug resistance phenotypes exhibited by the bacteria. Interestingly, the four isolates from HIV patients with integrons were resistant to cotrimoxazole. This may be because integrons normally carry a dfr gene that encodes trimethoprim resistance and a sul gene that encodes sulphonamide resistance which could be responsible for cotrimoxazole resistance 24.

The only isolate (*Serratia liquefaciens*) that produced AmpC was obtained from a healthy HIV negative individual. The isolate was resistant to ampicillin, penicillin, erythromycin, gentamicin, cefotaxime, cefoxitin and augmentin. AmpC beta-lactamase confers resistance to cephalothin, cefazolin, cefoxitin, most penicillins, and beta-lactamase inhibitor-beta-lactam combinations. AmpC enzymes are encoded by either chromosomal or plasmid-mediated genes in the Enterobacteriaceae, Most chromosomal AmpC β-lactamases can be found in *Enterobacter, Serratia, Pseudomonas, Acinetobacter and Citrobacter* spp 47. The presence of AmpC in this isolate may cause failure in cephalosporin treatment, which is considered to be effective in vitro. The presence of this organism may be due to selective pressure imposed by the indiscriminate use of antibiotics in this environment. This finding indicates that both healthy and sick individuals can serve as reservoirs of resistant bacteria in this environment.

## Limitation

The small number of isolates in our final analysis in spite of our sample size, which may not allow for a general conclusion to be made on the magnitude of antimicrobial resistance in the study group. Despite this limitation, our study shows the presence of multi-drug resistance in all the uropathogens recovered from the study group which requires guided prescriptions and rational use of antibiotics. Larger studies are needed to further investigate the magnitude of antimicrobial resistance in HIV patients.

## Conclusion

There is a higher burden of ASB among HIV positive individuals, predominantly among females. The implicated isolates are multi-drug resistant which requires guided prescriptions and rational use of antibiotics. There is a need for antibiotic stewardship programmes to control antibiotic resistance and prevent the spread of resistant organisms.

## Ethics of Study

Permission to conduct this research was obtained from the Research Ethics Committee of Obafemi Awolowo University Teaching Hospitals Complex, Ile-Ife, Nigeria. (Reference number: IRB/IEC/0004553).
